# Neurophysiological correlates of tactile width discrimination in humans

**DOI:** 10.3389/fnhum.2023.1155102

**Published:** 2023-05-12

**Authors:** Carla Pais-Vieira, Mehrab K. Allahdad, André Perrotta, André S. Peres, Carolina Kunicki, Mafalda Aguiar, Manuel Oliveira, Miguel Pais-Vieira

**Affiliations:** ^1^Centro de Investigação Interdisciplinar em Saúde (CIIS), Instituto de Ciências da Saúde (ICS), Universidade Católica Portuguesa, Porto, Portugal; ^2^Centre for Informatics and Systems of the University of Coimbra (CISUC), Coimbra, Portugal; ^3^Proaction Laboratory, Faculty of Psychology and Educational Sciences, University of Coimbra, Coimbra, Portugal; ^4^CINEICC, Faculty of Psychology and Educational Sciences, University of Coimbra, Coimbra, Portugal; ^5^Vasco da Gama Research Center (CIVG), Vasco da Gama University School (EUVG), Coimbra, Portugal; ^6^Center for Neuroscience and Cell Biology (CNC), Center for Innovative Biomedicine and Biotechnology (CIBB), University of Coimbra, Coimbra, Portugal; ^7^Department of Medical Sciences, iBiMED-Institute of Biomedicine, Universidade de Aveiro, Aveiro, Portugal

**Keywords:** tactile, discrimination, neurophysiology, EEG, width

## Abstract

**Introduction:**

Tactile information processing requires the integration of sensory, motor, and cognitive information. Width discrimination has been extensively studied in rodents, but not in humans.

**Methods:**

Here, we describe Electroencephalography (EEG) signals in humans performing a tactile width discrimination task. The first goal of this study was to describe changes in neural activity occurring during the discrimination and the response periods. The second goal was to relate specific changes in neural activity to the performance in the task.

**Results:**

Comparison of changes in power between two different periods of the task, corresponding to the discrimination of the tactile stimulus and the motor response, revealed the engagement of an asymmetrical network associated with fronto-temporo-parieto-occipital electrodes and across multiple frequency bands. Analysis of ratios of higher [Ratio 1: (0.5–20 Hz)/(0.5–45 Hz)] or lower frequencies [Ratio 2: (0.5–4.5 Hz)/(0.5–9 Hz)], during the discrimination period revealed that activity recorded from frontal-parietal electrodes was correlated to tactile width discrimination performance between-subjects, independently of task difficulty. Meanwhile, the dynamics in parieto-occipital electrodes were correlated to the changes in performance within-subjects (i.e., between the first and the second blocks) independently of task difficulty. In addition, analysis of information transfer, using Granger causality, further demonstrated that improvements in performance between blocks were characterized by an overall reduction in information transfer to the ipsilateral parietal electrode (P4) and an increase in information transfer to the contralateral parietal electrode (P3).

**Discussion:**

The main finding of this study is that fronto-parietal electrodes encoded between-subjects’ performances while parieto-occipital electrodes encoded within-subjects’ performances, supporting the notion that tactile width discrimination processing is associated with a complex asymmetrical network involving fronto-parieto-occipital electrodes.

## 1. Introduction

Tactile discrimination processing requires the integration of sensory, motor, and cognitive information in humans and animals (Chapman and Ageranioti-Bélanger, 1991; Krupa et al., 2004; O’Doherty et al., 2011; Simões-Franklin et al., 2011; Pais-Vieira et al., 2013a,^2015^; Adhikari et al., 2014; Kunicki et al., 2019). Width discrimination is a specific type of tactile information processing that has been extensively studied in rodents (Krupa et al., 2001, 2004; Wiest et al., 2010; Pais-Vieira et al., 2013a,b, 2015; Thomson et al., 2014), but remains largely undescribed in humans (Louw et al., 2002; Perrotta et al., 2020). Width discrimination in the sub-centimeter scale constitutes a critical survival skill for rodents, namely through the use of the vibrissae (Vincent, 1912; Carvell and Simons, 1990; Brecht et al., 1997). Meanwhile, width discrimination in humans is relevant for many activities (e.g., surgical procedures, sewing, etc.), but its role as a critical survival skill is debatable. Nonetheless, the large body of knowledge gathered in the latter species, can significantly inform us about the neural basis of somatosensory processing and lead to new lines of inquiry regarding this function in humans. For example, studies in rodents have revealed that whisker-dependent width discrimination performance is associated with widespread and dynamic interactions involving information transfer in theta, beta, and gamma frequency bands in a fronto-parieto-occipital network (Kunicki et al., 2019). These regions and frequency bands are known to play a relevant role in tactile processing of shape, texture, and electrical stimuli detection in humans (see, for example, Sathian, 2016; Alsuradi et al., 2022 for reviews), but their role in width discrimination remains unknown.

**FIGURE 1:**
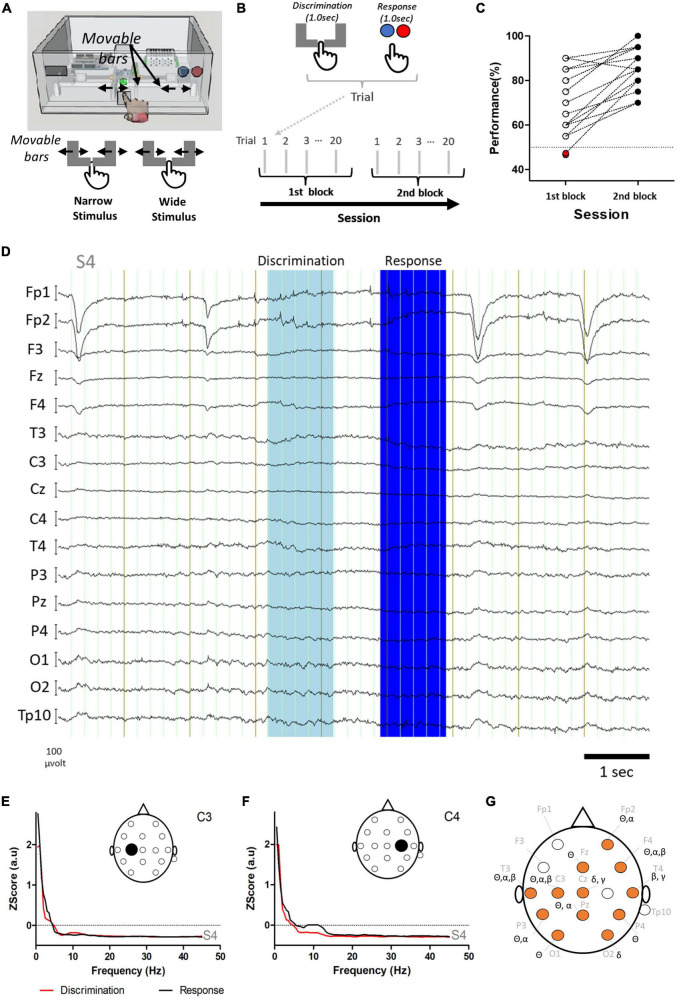
Study design, behavioral performance, and differences in power of frequency bands. **(A)** Tactile width discrimination task and representation of Narrow and Wide stimuli delivered to the index finger of the subject (i.e., passive tactile stimulation). In both Wide and Narrow stimuli, the bars touched the index finger. **(B)** In each trial, the discrimination period and the response period were analyzed. Sessions consisted of two blocks of 20 trials, each with 9–11 trials with the Narrow stimulus and 9–11 trials with the Wide stimulus. **(C)** An overall improvement in performance was observed between the first block (empty or red circles) and the second block (black filled circles). Red filled circles indicate two subjects that presented performances below chance. Note that some subjects presented the same performance and therefore one circle may correspond to more than one subject. **(D)** Example of raw electroencephalographic (EEG) activity recorded from subject S4 during the task. The discrimination and response periods are indicated in light and dark blue, respectively. **(E,F)** Power spectra in electrodes C3 **(E)** and C4 **(F)** for the discrimination (red) and response (black) periods of the EEG activity of subject S4 presented in D. **(G)** Comparison of power in the main frequency bands revealed an extensive network of electrodes with significant differences (orange circles) between the discrimination and the response periods (also see text and Table 1 for details). Note that for the gamma frequency band only the lower part of the spectra was analyzed (low gamma: 30.0–45.0 Hz).

To take advantage of the extensive body of knowledge existing in width discrimination in rodents, we have recently developed a width discrimination task for humans (Perrotta et al., 2020) that mimics the original rodent task (Krupa et al., 2001). In the version tested here (i.e., the passive version), subjects were required to insert the right index finger in a small chamber and wait for the tactile stimulus to be delivered by two movable non-visible bars (Figures 1A, B). In each trial, the bars moved toward the right index finger and pressed it, forming one of two widths: “Wide” or “Narrow.” The subject was then required to make a motor response in one of two pushbuttons, to indicate which stimulus was delivered. Preliminary data from a small number of subjects suggested that changes in the low gamma band may be relevant for tactile width discrimination (Perrotta et al., 2020). The present study is aimed at performing a more detailed and comprehensive analysis of the neurophysiological correlates of tactile width discrimination task.

Passive tactile width discrimination requires that subjects: (i) identify the presence of the stimulus (i.e., the bars touch the index finger), (ii) compare the relative width of the current stimulus with a previously experienced width (e.g., a previous experience of the Narrow stimulus), and (iii) make a motor response to indicate which stimulus was delivered. These actions are expected to engage neural circuits involving somatosensory processing, motor, as well as higher cognition processes such as working memory (Spitzer and Blankenburg, 2011) and attention (Bauer et al., 2006). Previous studies of tactile discrimination have explored EEG correlates of tactile processing using stimulation current, grating orientation, textures, or vibratory stimuli; due to their ability to generate EEG evoked responses. These studies have reported the engagement of a complex network of regions involving the somatosensory, pre-frontal, occipital, and the parietal cortexes, typically in alpha, mu, and beta frequency bands (Zangaladze et al., 1999; Pfurtscheller et al., 2001; Graimann et al., 2002; Palva and Palva, 2007; Spitzer and Blankenburg, 2011; Van Ede et al., 2011; Adhikari et al., 2014; Ishigaki et al., 2016; Moungou et al., 2016; Yao et al., 2016, 2017a,b; Genna et al., 2017, 2018; Whitmarsh et al., 2017; Eldeeb et al., 2020; Forschack et al., 2020; Su et al., 2020; Alsuradi et al., 2022). Namely, tactile discrimination seems to be associated with a recurrent network involving the somatosensory, parietal, occipital, and pre-frontal regions (Stilla et al., 2007; Adhikari et al., 2014) operating through beta (feedforward) and gamma (recurrent) frequency bands. In addition, clinical studies (Graimann et al., 2002) and studies involving brain-machine interfaces and tactile processing (Yao et al., 2016, 2017a,b), have showed that event related synchronization/desynchronization (ERS/ERD) in the alpha and beta frequency bands in the somatosensory cortex, are relevant for tactile and attention information.

Adhikari et al. (2014) demonstrated that neural networks associated with tactile processing operate on timescales of less than 200 ms. However, the passive tactile width discrimination task delivers stimuli through movable bars, which makes the duration of the tactile stimulus delivery longer (Perrotta et al., 2020). Therefore, even though tactile perception occurs as soon as the bar touches the finger, width discrimination will only occur after the complete stop of the bars movement and depends on the participant’s judgment that they will not move further (approximately 1 s). Due to this constraint, the identification of neural correlates of passive tactile width discrimination may benefit from analysis that are capable of detecting changes in neural activity occurring in relatively large timescales (i.e., hundreds of milliseconds to seconds).

Analysis of power and relative power in specific frequency bands have been previously used to analyze large time intervals (Drummond et al., 1991; Graimann et al., 2002; Gervasoni et al., 2004; Pereira et al., 2007; Yao et al., 2016, 2017a,b; Pais-Vieira et al., 2019). The use of relative power is a strategy widely used in the literature both in studies with human electrophysiology (Drummond et al., 1991; Moretti et al., 2009; Arns et al., 2013; Finnigan et al., 2016) and in animal models (Gervasoni et al., 2004; Pereira et al., 2007; Pais-Vieira et al., 2019). Pais-Vieira et al. (2019); adapted from Gervasoni et al. (2004) created maps comparing two ratios (low frequencies and high frequencies) that were used to identify transitions of neurophysiological states induced by pharmacological manipulation. These previous studies support the use of these ratios of power to describe broad neurophysiological states that may correlate to tactile width discrimination behaviors.

The present study had two aims. The first aim was to describe general changes occurring in neural activity during the periods of discrimination and response. Such description is relevant because it allows comparing neural activity in this task with that of previous reports. The second aim was to identify changes in neural activity that were relevant for the performance in the task within- and between-subjects. Such description is relevant for this and other tasks involving tactile processing, because it may help identifying and/or explaining the basis of differences in tactile performance between individuals and within the same individual. We hypothesized that: H1: EEG correlates of tactile width discrimination (power of frequency bands and/or relative power) change between the tactile stimulus discrimination and the motor response periods (Adhikari et al., 2014); H2: EEG power correlates to the behavioral performance between-subjects (interspecific) (Su et al., 2020) and within-subjects (i.e., between blocks of trials of the same subject–intraspecific).

To describe the neurophysiological correlates of tactile width discrimination in humans we have recorded EEG activity from subjects performing two blocks of a passive version of the task. The electrophysiological data recorded during the discrimination period was compared (power and relative power) to the response period, and the relative power was compared to the subjects’ performance (intra and interspecific). In addition, we also described the transfer of information between electrodes through the Granger causality analysis.

## 2. Materials and methods

### 2.1. Subjects

A total of 18 subjects asymptomatic for neurological and sensory motor disorders were initially tested. Half of the subjects studied were female (9/18 = 50.0% female; with 28.9 ± 7.28 years old; min: 18 years old, max: 40 years old). One subject (S17) completed both blocks, but technical problems did not allow using the neural data. Two subjects (S1 and S2) performed only one block with a total of 50 trials. Two subjects (S2 and S3) presented a performance below chance in the first block and therefore were not included in the analysis of correlation between relative power and width discrimination performance between-subjects (also see section “3 Results”). Due to experimental design, performance, or technical problems, in four subjects, only data from one block was collected and analyzed (S1, S2, S3, and S5). Fourteen subjects completed both blocks and had the neurophysiological data recorded (*N* = 14 sessions, total of 28 blocks). Three subjects (S1, S6, and S10) were tested a second time (in a separate day) in an easier version of the task to evaluate the neural correlates of width discrimination when the cognitive demands of the task were lowered (see section “2.1.1 Width discrimination task,” below). Due to these experimental design contingencies, the number of blocks and subjects analyzed in each experiment is indicated in each subsection.

#### 2.1.1. Width discrimination task

The width discrimination task consisted of a box with a front panel where the subject was required to insert the right index finger (Figure 1A). In each trial a yellow light in the front panel indicated that the subject should insert the finger. After inserting the finger, a computer vision algorithm, fed by the signal streamed from a camera placed behind the front panel, detected when the finger was in the appropriate position, switching the front panel light to green. Then, two movable bars pressed the index finger of the user forming a “Narrow” or “Wide” aperture in each trial. This period corresponded to the Discrimination. A timestamp for the Discrimination period was automatically calculated by the software and sent to a text file for posterior analysis. After the tactile stimulation, the bars moved to their initial position and the frontal panel light switched to red, to indicate that the subject should remove the index finger and press a pushbutton (indicating the type of stimulus delivered) (Figure 1B). When the subject pressed one of the pushbuttons, a second timestamp was automatically recorded by the software in the text file which indicated the Response period. For each subject the width of the finger was initially calculated and used to calibrate the bars. During the sessions, Narrow stimuli corresponded to –0.5 mm on each side of the finger and Wide stimuli corresponded to –0.3 mm. It should be noted that the absolute distance between Wide and Narrow varied for each subject because it depended on the index finger width.

To test the effects of task difficulty, three subjects (S1, S6, and S10) were tested in a separate day in an easier version of the task. In the easier version of the task the difference between the Wide and the Narrow stimuli was increased to a total of 2.0 mm in each side of the original measure for the right index finger. Each of these three subjects was tested in a session with two blocks of 20 trials (*N* = 3 sessions, total of six blocks).

The present study was approved by the Ethics Committee of the University of Minho (SECVS 148/2016); and the Comité para as Ciências da Saúde of the Catholic University of Portugal (39/2017), according to the Code of Ethics of the World Medical Association (Declaration of Helsinki) for experiments involving humans. All experiments were performed in accordance with relevant named guidelines and regulations. All participating subjects voluntarily filed an informed consent. All subjects were tested at the Catholic University of Portugal (subjects tested in the regular version of the task). The three subjects that were tested a second time (in the easier version of the task), were tested at the University of Minho.

### 2.2. EEG recordings and pre-processing

Electroencephalographic recordings were made from 16 electrode channels at 1,000 Hz using a 10–20 placement (V-Amp, actiCAP; Brain Products GmbH, Gilching, Germany). Signals were recorded using the Brain Vision Recorder (version 2.1.0, Brain Products, Gilching, Germany) and analyzed using Brain Vision Analyzer (version 2.2.1, Brain Products, Gilching, Germany) and Matlab (Mathworks, 2018b, Natick, MA, USA). Preprocessing included re-referencing using all channels as reference (Bertrand et al., 1985). A notch filter was applied (50 Hz). A zero-phase shift, 4th order Butterworth filter, with a low cutoff of 0.5 Hz and high cutoff of 70 Hz with a time constant of 0.3183 was used. Ocular correction was performed using the Gratton and Coles algorithm (already implemented in Visual Analyzer).

### 2.3. EEG power analysis

The data was then segmented according to the discrimination and response markers generated by the tactile discrimination task software, with a window of 1,000 ms (–500 up to 500 ms after each marker). The Fast Fourier Transform with a resolution of 0.5 Hz was then applied.

As the number of electrodes used in the present study does not allow for source analysis, the present findings are presented and discussed regarding the position of each electrode. The activity from the electrode C3 was included in the analysis of information transfer due to its location above the somatosensory cortex. Therefore, even though the activity from this electrode did not present a significant correlation with task performance (see section “3 Results”), it was considered as potentially relevant for the understanding of the neurophysiological correlates of tactile width discrimination.

Power was analyzed in terms of the frequency bands: delta (0.5–4.5 Hz), theta (4.5–8.5 Hz), alpha (8.5–13.5 Hz), beta (13.5–30.5 Hz), and low gamma (30.5–45 Hz). Data was only analyzed up to 45 Hz (described here as low gamma frequency band), to match the state map values used in a previous study (Pais-Vieira et al., 2019). Z scores were then calculated, and the different periods (discrimination and response) were compared. Comparison of power was made between the two periods, instead of using a baseline, because we have observed in a previous study that large intertrial intervals quickly led to mental exhaustion of participants.

Relative power analysis was performed according to an adaptation to the method described by Gervasoni et al. (2004) where two ratios are calculated based on the average power of higher and lower frequencies. The analyses of ratios 1 and 2 are not mutually exclusive, since the lower frequencies are used to calculate both ratios; namely: ratio 1, R1 = (0.5–20 Hz)/(0.5–45 Hz) and ratio 2, R2 = (0.5–4.5 Hz)/(0.5–9 Hz). According to the original authors, these intervals have previously been chosen based on their ability to separate different behavioral states. It is noteworthy that, as the values of power have been normalized across subjects, the ratios may appear as positive or negative values. These ratios were then used either in combination as a coordinate (Ratio 1: abscissa, Ratio 2: ordinate) to define state maps or otherwise compared to other relevant variables (e.g., performance).

#### 2.3.1. Power comparison during discrimination and response periods

Analysis of power and analysis of relative power between the discrimination (±500 ms) and response (±500 ms) periods was tested using permutations tests. The description of the proportion of electrodes that presented significant changes between the discrimination and the response periods was calculated from the total of electrodes recorded (*N* = 16). The description of simultaneous changes occurring in ratio 1 and ratio 2 was done using state maps where vectors indicated the size and direction of change. For this the values of ratio 1 and ratio 2 during the discrimination period were used as coordinates of the arrow tail (Ratio 1: X-axis, and Ratio 2: Y-axis in the first block). Meanwhile, the values of ratio 1 and ratio 2 during the discrimination period were used as the coordinates of the arrowhead (Ratio 1: X-axis, and Ratio 2: Y-axis in the second block).

#### 2.3.2. Correlation of relative power with task performance

Ratios of power for higher and lower frequency bands were used to study neurophysiological correlates of tactile discrimination performance. A total of *N* = 29 blocks in 16 subjects were analyzed. Two blocks with performances below chance were not analyzed here. For this, ratio 1 and ratio 2 values were separately compared to the performance of each subject in each block using Spearman’s Rho correlation.

#### 2.3.3. Comparison of power and performance between blocks

Changes in ratios of power and changes in performance between blocks were separately compared for each ratio. First, the difference in power for ratio 1 in the second and the first block was calculated (Diff Ratio 1). Second, the difference in performance between the second and the first block was calculated (Diff Performance). Third, the absolute (i.e., modulus) value of the ratio 1 difference between the second and the first block was calculated [Abs (Diff Ratio 1)]. Lastly, a Spearman correlation was calculated between the absolute (i.e., modulus) value of the ratio 1 difference between the second and the first block, and the difference in performance between the second and the first block. The same calculations were then performed for ratio 2.

### 2.4. Granger causality

Granger causality was used to investigate directional connectivity between different channels (Granger, 1969). Only the electrodes that presented significant correlation with task performance (Fp2, F4, T4, P3, P4, and O2–see section “3 Results”), and in addition electrode C3 (due to its location above the somatosensory cortex) were tested. Signals were first filtered with a Butterworth filter (0.5–45 Hz) with a linear envelop of 2 Hz and with a zero-phase filter (Matlab function “filtfilt”). Signals were then tested in both directions (e.g., channel A prediction of channel B and vice-versa) using the Matlab function “gctest” with a two-millisecond lag.

The alpha value of the Granger causality test was corrected for the total number of comparisons using False Discovery Rate (FDR). Analysis of significant causality changes in specific channels (i.e., appearing or disappearing between the first and the second blocks) was performed only for sessions with EEG data for the two blocks. The panels with arrows connecting different electrodes were calculated using the number of subjects that presented significant Granger causality tests between pairs of electrodes.

### 2.5. Statistical analysis

Results are presented as Mean and standard deviation (Mean ± SD). Exact permutation tests were used (function “permutation Test” in Matlab) to compare the power in each frequency band between the two different periods, to compare power between different tactile stimuli, and to compare within-subjects neurophysiological changes (i.e., between the first and the second blocks). Spearman’s Rho was used to correlate subjects’ performances with the power of frequency bands. This analysis was followed by Benjamini and Hochberg (1995) correction for false discovery rate (Bertrand et al., 1985). Paired samples *t*-tests were used to compare the behavioral performance. The corrected *P*-values indicating the corrected statistic are presented in the text immediately after the original *P*-values. Significance was considered for alpha at 5%.

## 3. Results

### 3.1. Behavioral results

The behavioral performance in the task was 76.55% ± 14.32 and the response latency was 1.045 ± 0.266 s. Response latency was not correlated to performance (Rs = 0.1277, *P* = 0.5091, corrected *P* = 0.8265). For the fourteen subjects that completed both blocks, and overall improvement was found between the first and the second block (first block: 69.81 ± 14.31% correct; second block: 85.36 ± 9.5%; Paired samples *t*-test: *t* = 5.315; df = 13, *P* = 0.0001) (Figure 1C).

### 3.2. Distinct task periods are associated with differences in power

Neural recordings were characterized by changes occurring in the power of different frequency bands in specific electrodes during the task. Examples of raw EEG activity and power spectra for the discrimination and response periods in the electrodes recording above the primary somatosensory cortex (C3, C4) are presented in Figures 1D–F. Analysis of power in delta (0.5–4.5 Hz), theta (4.5–8.5 Hz), alpha (8.5–13.5 Hz), beta (13.5–30.5 Hz), and low gamma (30.0–45 Hz) bands indicated that the periods of discrimination (i.e., stimulus delivery) and response (i.e., choosing and pressing one button) were characterized by fundamentally different patterns of activity throughout the network of electrodes recorded (Figure 1G). Table 1 presents the statistically significant results for the comparison between the discrimination and response periods for each frequency band in each electrode. An overall reduction in power was present, frequently in more than one frequency band. The exceptions to this were electrodes Fp1, F3, C4, T4, Pz, and TP10; where no differences were found between the two periods analyzed for any frequency band. For channels with significant changes between the two periods, the theta frequency band was the most frequently affected (8/16 = 50% of the electrodes), followed by alpha and beta frequency bands (each with 4/16 = 25% of electrodes). Lastly, delta and low gamma bands (2/16 = 12.5% of electrodes) were less often affected. These results indicated that the discrimination and the response periods were associated, with a bilateral asymmetrical distribution of changes in power, occurring most commonly in theta, but also in other frequency bands in a network of electrodes recording from frontal, temporal, parietal, and occipital regions.

**TABLE 1:** Differences in power during the discrimination and response periods.

		Discrimination		Response			
		**Mean**	**Standard deviation**	**Mean**	**Standard deviation**	***P*-value**	**Significant**
Alpha	FP2	0.0302	0.1911	-0.0452	0.0170	0.0048	**
Theta	FP2	0.1280	0.3992	-0.0298	0.0263	0.0001	***
Theta	FZ	0.5302	0.5491	0.2965	0.3321	0.0435	*
Theta	F4	0.1831	0.3338	0.0488	0.1714	0.0443	*
Alpha	F4	0.0251	0.0895	-0.0320	0.0423	0.0012	**
Beta	F4	-0.0525	0.0269	-0.0710	0.0202	0.0025	**
Theta	T3	0.2493	0.3304	0.0213	0.0924	0.0001	***
Alpha	T3	0.1073	0.1798	-0.0266	0.0428	0.0000	***
Beta	T3	0.0018	0.1207	-0.0540	0.0307	0.0070	**
Theta	C3	0.3643	0.6296	0.0948	0.2123	0.0128	*
Beta	C3	-0.0274	0.0780	-0.0545	0.0173	0.0414	*
Delta	CZ	2.6802	0.8070	2.1956	0.7449	0.0187	*
Gamma	CZ	-0.1098	0.0440	-0.0846	0.0392	0.0213	*
Theta	P3	0.7042	0.6632	0.1755	0.2201	0.0001	***
Alpha	P3	0.2103	0.2490	0.0186	0.0959	0.0000	***
Gamma	P3	-0.1159	0.0478	-0.0902	0.0301	0.0148	*
Theta	P4	0.7833	0.8218	0.3883	0.4634	0.0230	*
Beta	P4	-0.0457	0.0634	-0.0713	0.0282	0.0460	*
Theta	O1	0.7686	0.7061	0.3858	0.5206	0.0143	*
Delta	O2	2.8007	0.7517	2.3577	0.8418	0.0351	*
	Power values are presented as Z scores						

Statistically significant differences between the width discrimination and the motor response periods were found in multiple electrodes. Theta, Alpha, and Beta bands were most often significantly different between the two periods. Values are presented as Mean ± Standard Deviation. Note that power was normalized across subjects and therefore Z scores may be positive or negative (also see section “2 Materials and methods”).

*, **, and *** indicate P < 0.05, P < 0.01, and P < 0.0001, respectively.

**FIGURE 2:**
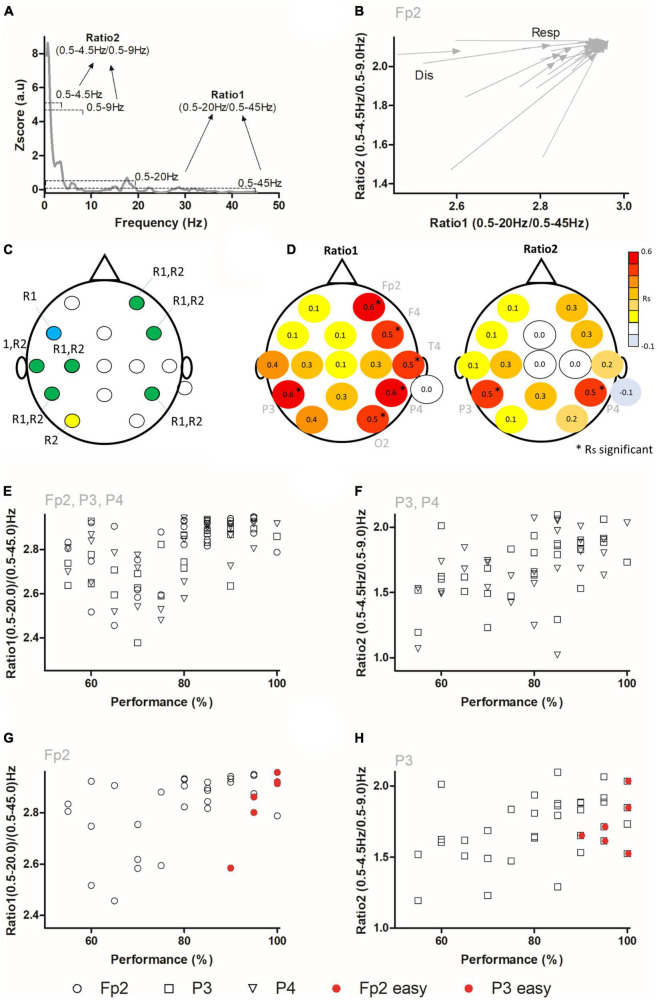
Ratios of frequencies are correlated to tactile performance. **(A)** State maps were calculated using two different ratios (Ratio 1 and Ratio 2). **(B)** Example of state map dynamics in Fp2 electrode during the discrimination and response periods. The arrow tail indicates the ratios coordinates (ratio 1 in abscissa axis and ratio 2 in ordinate axis) during the discrimination period (Dis), and arrow tip indicates the coordinates of ratios during the response period (Resp). An overall displacement toward higher ratio 1 and ratio 2 was observed for this electrode. **(C)** Network of electrodes with significant differences for ratio 1 (Blue), ratio 2 (yellow), or both (green). **(D)** Correlation between performance and neural activity during the discrimination period. Red and yellow indicate positive correlations, while blue indicates negative correlations. Asterisks indicate significant Spearman correlations. On the left panel, Rs (Spearman’s Rho) correlation values between ratio 1 and performance are presented. Ratio 1 was significantly correlated to tactile width performance in electrodes positioned in frontal (Fp2, F4), parietal (P3 and P4), temporal (T4), and occipital regions (O2). On the right panel the Spearman correlation values between ratio 2 and performance are presented. Ratio 2 was correlated to tactile performance in P3 and P4 electrodes. **(E)** Example of individual values of ratio 1 and performance for electrodes Fp2, P3 and P4. **(F)** Example of individual values of ratio 2 and performance for electrodes P3 and P4. **(G,H)** The same data as in panels **(E,F)** is presented, but only for one channel. The six red circles show ratio 1 values in channel Fp2 **(G)** and for ratio 2 in channel P3 **(H)** for three additional subjects (two blocks per subject) tested in an easier version of the task. For panels **(G,H)** (after statistical analysis), data points that were superimposed in the graph, were increased by 0.2 units in the X axis to facilitate visualization.

Due to the large number of differences in power found in multiple electrodes and frequency bands, we then set to analyze changes occurring simultaneously in multiple frequency bands across the scalp. For this, an analysis of state maps composed by ratios of frequency bands was performed (Gervasoni et al., 2004; Pais-Vieira et al., 2019; Figures 2A–H). It has been previously shown that analysis of ratios of power accurately captures global cortical dynamics and/or associated behaviors and therefore it is useful when multiple changes in multiple frequency bands and electrodes occur, as well as when large time intervals are to be analyzed (Drummond et al., 1991; Gervasoni et al., 2004; Moretti et al., 2009; Arns et al., 2013; Finnigan et al., 2016; Pais-Vieira et al., 2019). As depicted in Figure 2A; ratio 1 (used to analyze changes occurring in higher frequency bands), was calculated as (0.5–20 Hz)/(0.5–45 Hz) and ratio 2 (used to analyze changes occurring in lower frequency bands) was calculated as (0.5–4.5 Hz)/(0.5–9 Hz). In Figure 2B, an example of the state maps for channel Fp2 during the discrimination (Dis) and response (Resp) periods is presented. The arrow tail corresponds to the ratio 1 and ratio 2 coordinates during the discrimination period, while the arrowhead corresponds to the ratio 1 and ratio 2 coordinates during the response period. The displacement of each vector reflects the neural dynamics occurring as the subject moved from a state associated with the discrimination (lower left side) to the neural state corresponding to response (upper right side).

The analysis of these ratios (Table 2) revealed that the discrimination and response periods were characterized by different ratios of frequencies throughout a network involving multiple electrodes. Namely, significant differences were found for ratio 1 in electrodes Fp2, F3, F4, T3, C3, P3, and P4. Meanwhile, for ratio 2, significant differences were found for electrodes Fp2, F4, T3, C3, P3, P4, and O1, with a near significant value for Pz (*P* = 0.0506, n.s.). A visual summary of these findings is depicted in Figure 2C, where the network of electrodes associated with significant differences in either or both ratios during the discrimination and response periods is presented. These results demonstrated that ratios of frequencies in a network involving frontal-temporal-parietal-occipital electrodes encoded different periods of the width discrimination task.

**TABLE 2:** Differences in ratios of frequency bands during the discrimination and response periods.

	Ratio 1						Ratio 2					
	**Discrimination**		**Response**				**Discrimination**		**Response**			
	**Mean**	**Standard deviation**	**Mean**	**Standard deviation**	***P*-value**	**Significant**	**Mean**	**Standard deviation**	**Mean**	**Standard deviation**	***P*-Value**	**Significant**
Fp1	2.820	0.140	2.007	0.126	0.419	–	2.876	0.126	2.053	0.109	0.334	–
**Fp2**	**2.819**	**0.140**	**2.004**	**0.161**	**0.0011**	**	**2.920**	**0.071**	**2.105**	**0.028**	**<0.0001**	***
**F3**	**2.813**	**0.153**	**2.000**	**0.089**	**0.0148**	*	2.894	0.078	2.038	0.120	0.3156	–
Fz	2.838	0.107	1.847	0.167	0.3983	–	2.867	0.071	1.913	0.212	0.1866	–
**F4**	**2.856**	**0.105**	**1.976**	**0.132**	**0.0201**	*	**2.913**	**0.049**	**2.065**	**0.062**	**0.0084**	**
**T3**	**2.614**	**0.336**	**1.918**	**0.151**	**0.0014**	**	**2.842**	**0.160**	**2.069**	**0.060**	**0.0001**	***
**C3**	**2.736**	**0.208**	**1.877**	**0.271**	**0.0130**	*	**2.860**	**0.119**	**2.025**	**0.124**	**0.0046**	**
Cz	2.882	0.057	1.873	0.230	0.0631	–	2.909	0.049	1.963	0.239	0.1516	–
C4	2.739	0.312	1.846	0.287	0.3527	–	2.830	0.151	1.943	0.183	0.2055	–
T4	2.612	0.456	1.958	0.246	0.1837	–	2.764	0.267	2.055	0.112	0.0656	–
**P3**	**2.779**	**0.140**	**1.702**	**0.240**	**0.0213**	*	**2.868**	**0.085**	**1.974**	**0.113**	**<0.0001**	***
Pz	2.879	0.071	1.828	0.263	0.8452	–	2.890	0.067	1.967	0.113	0.0506	–
**P4**	**2.793**	**0.144**	**1.697**	**0.279**	**0.0177**	*	**2.877**	**0.063**	**1.899**	**0.175**	**0.0259**	*
**O1**	2.505	0.341	1.657	0.270	0.1136	–	**2.674**	**0.168**	**1.857**	**0.230**	**0.0070**	**
O2	2.550	0.323	1.744	0.170	0.2726	–	2.667	0.254	1.833	0.305	0.6303	–
Tp10	2.797	0.142	1.982	0.115	0.3246	–	2.866	0.101	2.027	0.157	0.5451	–

An overall increase in ratio 1 and ratio 2 was observed between the discrimination and the response periods. This increase occurred with an asymmetrical pattern and across multiple electrodes.

A near significant value was found for ratio 2 in the Pz electrode.

Bold values indicate statistical significance.

*, **, and *** indicate *P* < 0.05, *P* < 0.01, and *P* < 0.0001, respectively.

### 3.3. Ratios of power predict performance

We then analyzed if the ratios of power for higher and lower frequency bands could be associated with the performance in the width discrimination task (*N* = 29 blocks in 16 subjects; two blocks with performances below chance were not analyzed here). For this, ratio 1 and ratio 2 values were compared to the performances in each session using Spearman’s Rho correlation (i.e., the value of the session was compared to the overall session ratio 1 and ratio 2 values). As presented in Figure 2D, ratio 1 (left panel) and ratio 2 (right panel), significantly correlated to subjects’ performance in a bilateral asymmetrical network across the scalp. For ratio 1, significant Spearman’s Rho correlations were present in electrodes Fp2 (Rs = 0.5545, *P* = 0.0018, corrected *P* = 0.0192), F4 (Rs = 0.5285, *P* = 0.0023, corrected *P* = 0.02048), T4 (Rs = 0.5007, *P* = 0.0057, corrected *P* = 0.026057), P3 (Rs = 0.5796, *P* = 0.001, corrected *P* = 0.032), P4 (Rs = 0.5647, *P* = 0.0014, corrected *P* = 0.0224), and O2 (Rs = 0.489, *P* = 0.0071, corrected *P* = 0.0284). Meanwhile, for ratio 2 significant Spearman’s Rho correlations were present for electrodes P3 (Rs = 0.5416, *P* = 0.0024, corrected *P* = 0.0192) and P4 (Rs = 0.5094, *P* = 0.0048, corrected *P* = 0.0256). Examples of these correlations are presented in Figures 2E, F. These results indicated that the ratios of higher and lower frequencies reflected the overall performance in the task in a network associated with electrodes Fp2, F4, T4, P3, P4, and O2.

To determine if these correlations would still be present when task difficulty was changed, we specifically manipulated the difficulty of the task (see section “2 Materials and methods” for details) and performed three additional sessions in three subjects. These subjects, that had already been previously tested in this task, performed each one a session with two blocks in an easier version of the tactile task while EEG recordings were performed. For this the difference between the Wide and the Narrow stimuli was increased by 4.0 mm. Under such conditions the behavioral performance was of 96.67 ± 4.08%, indicating an almost perfect performance from all three subjects. We then reanalyzed state maps comparing the ratios of frequency bands, but now included the six new blocks from the sessions performed in the easier version of the task. Examples of these state maps are presented in Figures 2G, H (ratio 1 Fp2 and ratio 2 P3, respectively) where the red filled circles represent these six additional blocks. Comparison of the ratios of frequencies and the performance, when these six blocks were pooled with the remaining sessions and analyzed, still generated significant correlations for channels Fp2 (Figure 2G), F4, and P4 in ratio 1 (Fp2: Rs = 0.4671, *P* = 0.0047, corrected *P* = 0.0376; F4: Rs = 0.4422, corrected *P* = 0.0073, corrected *P* = 0.0292; P4: Rs = 0.4232, *P* = 0.0113; corrected *P* = 0.0226), but not for T4, P3, and O2 (T4: Rs = 0.1202, *P* = 0.4915, corrected *P* = 0.4915, n.s.; P3: Rs = 0.1711, *P* = 0.3257, corrected *P* = 0.3722, n.s.; O2: Rs = 0.3139, *P* = 0.0663; corrected *P* = 0.0884, n.s.). Meanwhile, a significant correlation between the performance and ratio 2 in electrode P3 was maintained (P3: Rs = 0.4457, *P* = 0.0073, corrected *P* = 0.0292) (Figure 2H), but not for electrode P4 (P4: Rs = 0.3334, *P* = 0.0504, corrected *P* = 0.08064, n.s.). These results suggested that ratios of frequency bands from electrodes Fp2, F4, P3, and P4 predicted width discrimination performance independently of the degree of difficulty.

### 3.4. Dynamics of frequency bands predict task performance

**FIGURE 3:**
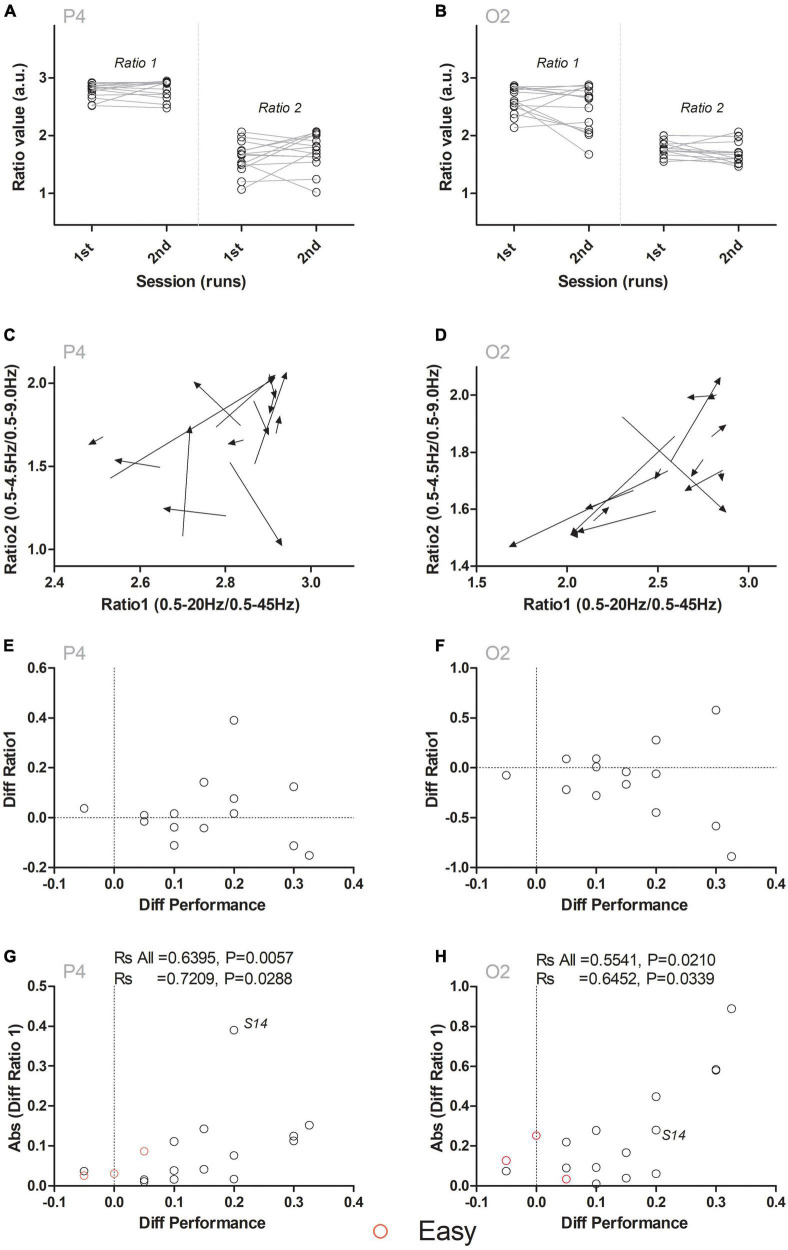
Ratio 1 dynamics reflect the changes in performance within-subjects. **(A,B)** Depict ratio1 and ratio 2 values for the first and the second block in channels P4 and O2. No differences between ratio 1 or ratio 2 were observed between the first and second block. **(C,D)** Simultaneous analysis of ratio 1 and ratio 2 suggested an overall pattern of change, especially in ratio 2 for electrode O2, associated with the lower right quadrant of the panel. **(E,F)** Changes in ratio 1 between the first and the second blocks (Diff Ratio 1) were compared to the changes in performance (Diff Performance) between the first and the second blocks. Analysis of the distribution of data did not reveal a consistent increase or decrease between the two blocks. Instead, a consistent departure from the value 0 (i.e., an increase in the distance to 0) occurred as the difference in performance increased. This generated an overall symmetrical pattern around the value of 0 for the Y axis, especially for electrode O2. **(G,H)** To analyze this symmetrical pattern, the absolute (i.e., modulus) value of the difference [Abs (Diff Ratio 1)] was calculated for channels P4 **(G)** and O2 **(H**). Performing a Spearman correlation analysis indicated that the Abs (DiffRatio1) predicted changes in performance (Diff Performance) between the first and the second blocks. Red open circles indicate six additional blocks (*N* = 3 sessions in three subjects), where an easier (“Easy”) version of the task was used. The overall correlation between ratio 1 dynamics and changes in performance was maintained for channels P4 and O2. “Rs All” indicates Spearman’s Rho when blocks from subjects performing the easier version of the task were pooled with the remaining blocks.

Having determined that the network of regions comprising Fp2, F4, P3, and P4 predicted the performance in the task, we then asked if the dynamics of fronto-parietal-temporal-occipital network ratios could also predict the changes in performance within-subjects (i.e., between the first and second blocks of the session). In other words, we tested if the changes in the ratios from this network reflected the dynamics of improvements in performance for each subject. For this, we reanalyzed the data in electrodes Fp2, F4, P3, and P4 from the subsample of subjects that completed both blocks (*N* = 14 subjects in 28 blocks). The sequence of steps used to perform this analysis is detailed in Figure 3. First, we calculated the changes occurring in ratio 1 and ratio 2 in the same subject. This is depicted in Figures 3A, B, where the changes occurring in ratio 1 and ratio 2 of electrodes P4 (panel A) and O2 (panel B), between the first and the second blocks of the session are presented. Second, we analyzed both ratios simultaneously. In Figures 3C, D, both coordinates were arranged to form the arrow tail (Ratio 1: X-axis, and Ratio 2: Y-axis in the first block) and the arrowhead (Ratio 1: X-axis, and Ratio 2: Y-axis in the second block) of a vector. While no clear pattern could be observed in the P4 electrode (Figure 3C), an overall shift toward the right lower part of the coordinates space could be identified in O2 (Figure 3D). This suggested that changes in ratios (and especially in electrode O2) could be related to the differences in performance observed between the first and the second blocks. Third, the difference between neural activity in the two blocks was plotted in the ordinate axis (Diff Ratio) and the difference in performance in the two blocks was plotted in the abscissa axis (Diff Performance). This allowed comparing the physiological and behavioral evolution throughout the two blocks that composed the session. In Figures 3E, F, show the relation between difference in ratio 1 and the difference in performance between the two blocks. Even though no clear correlation could be observed between these two variables, an overall increase in the variance of ratio 1 (i.e., in the ordinates axis) was present, as the difference in performance increased (i.e., abscissa values increased). In other words, although no clear increase or decrease was observed in Diff Ratio 1 between the two blocks, the distance of Diff Ratio 1 to 0 in the Y axis increased as the Diff Performance increased, suggesting that Diff Performance could be correlated to the absolute (i.e., modulus) value of Diff Ratio 1. To explain this variation, we then tested if the symmetrical pattern observed in ratio of frequencies for the electrode O2 (Figure 3F) converted to its absolute values [Abs (Diff Ratio 1)] (which now reflected “a change in ratio 1 magnitude” instead of “an increase” or “a decrease” in ratio 1 magnitude) could explain the apparent pattern observed in the data. As presented in Figures 3G, H, a significant correlation was found between the dynamics of ratio 1 for electrodes P4 (P4 Ratio 1: Rs = 0.7209, *P* = 0.0036, corrected *P* = 0.0288; note that this result is also significant if the same analysis is performed without subject S14) and O2 (O2 Ratio 1: Rs = 0.6452, *P* = 0.0127, corrected *P* = 0.0339), but not for Fp2 (Ratio 1: Rs = 0.1424, *P* = 0.6273, corrected *P* = 0.73185, n.s.), F4 (F4 Ratio 1: Rs = -0.02225, *P* = 0.9398, corrected *P* = 0.9398, n.s.), T4 (T4 Ratio 2: Rs = 0.3471, *P* = 0.2241, corrected *P* = 0.5229, n.s.), P3 (P3 Ratio 2: Rs = -0.1891, *P* = 0.5173, corrected *P* = 0.7242, n.s.) or P4 (P4 Ratio 2: Rs = 0.2937, *P* = 0.3082, corrected *P* = 0.53935, n.s.). Therefore, changes in ratio 1 of electrodes P4 and O2 predicted the tactile width performance changes occurring between the first and second blocks (within-subjects).

To further test if these dynamics were independent of the level of difficulty in the task, we again analyzed the changes occurring in the absolute (i.e., modulus) difference of ratio 1 for electrodes P4 and O2 when the six blocks from the three sessions performed in the easier version of the task were included. As presented in Figures 3G, H with red open circles, the inclusion of these additional blocks still presented a significant correlation for P4 (Rs All = 0.6395, *P* = 0.0057) and for O2 (Rs All = 0.5541, *P* = 0.0210). These results indicated that the magnitude of the changes in dynamics of ratio 1 for electrodes P4 and O2 encoded the changes in performance in the behavioral task between the two different blocks of the same session.

### 3.5. Bidirectional Granger causality in electrode networks

Having identified that specific networks of electrodes were associated with the different periods of the task, and that they predicted tactile width performance, we then asked how information was transferred between these electrodes. For this, we performed two analysis of neural activity during the discrimination period using Granger Causality (GC). In the first analysis, GC tests were calculated for each pair of electrodes of the Fp2-F4-C3-T4-P3-P4-O2 network for all blocks. In the second analysis, the differences between the first and the second blocks were compared. This allowed studying the connectivity in pairs of the network of electrodes by identifying: (i) the direction of GC between two electrodes (electrode A to electrode B, electrode B to electrode A, or bidirectional), (ii) the network of electrodes associated with GC, (iii) if this network was associated with improved performances, and (iv) if the network of electrodes changed between the first and the second blocks.

**FIGURE 4:**
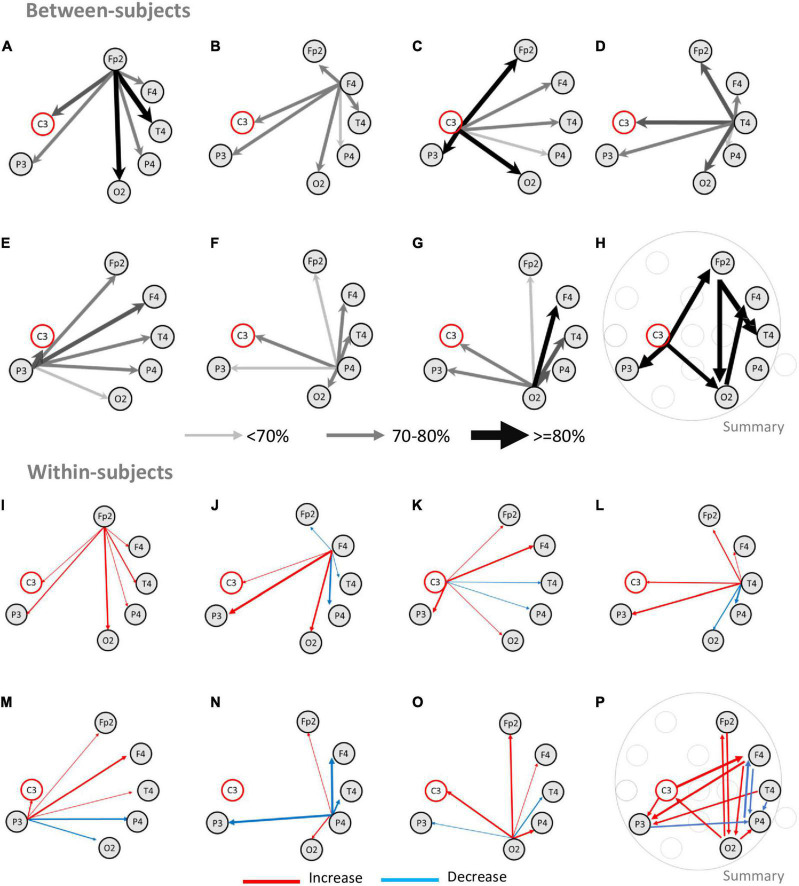
Information transfer networks during width discrimination. The upper half of the figure depicts the results from the analysis of Granger causality tests performed independently for each of the blocks analyzed (“between-subjects”). The lower half of the figure depicts the comparison between the first and the second blocks for each subject (“within-subjects”). Analysis of Granger causality was performed only for the C3 electrode and for the electrodes that presented significant correlation with performance. **(A–G)** The arrows depict the proportion of significant Granger causality tests. Wider and darker arrows indicate an increased fraction of blocks with a significant Granger causality test between two electrodes. Thinner and brighter arrows indicate a decreased fraction of significant Granger tests. The largest number of significant Granger causality tests occurred from the electrode Fp2 to the electrode T4 (90% of the blocks analyzed) and O2 (80% of the blocks) **(A)**, followed by the electrode C3 sending information to electrodes Fp2, P3, and O2 in 80% of the blocks **(C)**, and also electrode O2 sending information to electrode F4 (80% of the blocks) **(G)**. Electrode P4 generally presented the smallest number of significant tests. **(H)** Summary of the main significant connections for the network of electrodes present in at least 80% of the blocks. The pattern of connectivity most common between electrodes was C3 electrode sending information to electrodes Fp2 (80%), P3 (80%), and O2 (80%); Fp2 electrode sending information to electrodes O2 (80%) and T4 (90%), and lastly, electrode O2 sending information to F4 (80%). **(I–O)** The arrows depict the changes in the number of significant Granger causality tests between two blocks of the same subject. The wider arrows with increased color saturation represent the fraction of subjects with an increase (red) or decrease (blue) in significant Granger causality between the two blocks. **(I–O)** Changes in improvement between blocks were characterized by increases or decreases in information in specific pairs of electrodes that led to networks of information transfer that were closer to the network of information transfer for tactile width processing. The main changes observed involved increasing information transfer between electrodes Fp2-O2 (bidirectionally), F4-P3 (bidirectionally), C3-F4, and C3-P3 (bidirectionally). Decreases in information most often involved electrode bidirectional decreases of information transfer between P4 and P3, F4, and T4. **(P)** Summary of the main significant connections for the information transfer network predicting improvement between different subjects highlighting increases in C3, Fp2, F4, T4, P4 and O2; and decreases in information transfer between P4 and F4, T4, and P3.

We started by analyzing all blocks from the subsample of subjects that completed both blocks (*n* = 14 subjects, 28 blocks), to determine if information transfer during the discrimination period was associated with specific pairs of electrodes (also see Supplementary Table 1). The upper half of Figures 4A–H depicts the results of this analysis for all blocks (“between-subjects”). In Figures 4A–G, the size and the color of the arrows indicate the number of blocks where significant GC was found. Wide and dark arrows indicate pairs of electrodes where a large fraction of blocks presented significant GC tests, while a narrow light gray arrow indicates a significant GC test in a small number of blocks. Although all pairs presented bilateral connections (i.e., significant GC tests were found at least once for each pair of electrodes in both directions), most arrows were most often asymmetrical indicating asymmetrical information transfer between two electrodes.

As depicted in Figures 4A–G, significant GC tests were found across multiple electrodes with an increased number of blocks presenting significant GC causality between electrodes Fp2, F4, C3, T4, P3, and O2. The largest fraction of blocks was found for the pair Fp2 -T4 (Figure 4A; 90% of the blocks). Electrode pairs FP2-O2 (Figure 4A), C3-Fp2 (Figure 4C), C3-P3 (Figure 4C), C3-O2 (Figure 4C), and O2-F4 (Figure 4G) each presented significant GC tests in 80% of the blocks. Other electrode pairs presented smaller fractions of significant GC tests. This analysis revealed that, during width discrimination, information was transferred bidirectionally and asymmetrically between all pairs of electrodes studied, but more often from electrodes C3, Fp2, and O2 to electrodes Fp2, P3, O2, F4, and T4 (as summarized in Figure 4H).

We then tested if the pairs of electrodes that transferred information in the first block were the same that transferred information in the second block. For this the pairs of electrodes with significant GC tests in the first block for each subject were subtracted to the pairs of electrodes with significant GC tests in the second block (*N* = 14 subjects, 28 blocks). The lower half of Figures 4I–P presents this analysis (“within-subjects”). An increase in the number of significant GC tests for pairs of electrodes between the first and the second block are represented with a red arrow, while a decrease in the number of significant GC tests for pairs of electrodes between the first and the second block are represented with a blue arrow. The size and color saturation of the arrow indicate the number of significant tests (also see Supplementary Table 2 for details). The largest increases occurred between Fp2-O2 (bilaterally) (Figures 4I, O), F4-P3 (bilaterally) (Figures 4J, M), C3-P3 (Figures 4K, M), C3-F4 (Figures 4J, K), T4-P3 (Figures 4L, M), F4-O2 (Figures 4J, O). The largest decreases occurred between P3-P4 (bilaterally) (Figures 4M, N) and P4-F4 (bilaterally) (Figures 4J, N). It is noteworthy that the analysis of the decreases in GC tests between the first and the second blocks, indicated that the electrode P4 presented the largest decreases. These decreases, occurred for multiple electrode pairs, namely F4-P4 (bilaterally) (Figures 4J, N), T4-P4 (Figures 4L, N) (bilaterally), P3-P4 (bilaterally) (Figures 4M, N). The electrodes F4, T4, and P3 also presented decreases in the number of blocks with significant GC tests, but these numbers were smaller.

Overall, the increases in pairs of electrodes exchanging information tended to reflect changes that led to the pattern revealed by the analysis of all blocks (Figure 4H), where C3, Fp2, and O2 sent information to Fp2, F4, T4, P3, and O2; with some additional pairs appearing (e.g., O2-C3, F4-P3), while the decreases in pairs exchanging information more often involved F4, T4, P3, and P4. A summary of the increases and decreases in GC tests between the first and second blocks of each session is presented in Figure 4P.

## 4. Discussion

This study described neural correlates during tactile width discrimination in humans allowing: (1) a global description of differences in neural activity during the discrimination and response periods, and (2) identifying specific neural substrates of differences within-subjects and between-subjects in width discrimination performance. Subjects were able to discriminate between the two widths, with performances generally improving between the first and the second blocks. Neural activity in the period of tactile stimulation and the period of behavioral response was characterized by multiple changes in power across frequency bands of the electrodes recorded, as well as in ratios comparing power in higher and lower frequencies. Comparison of behavioral performances and ratios of frequency bands revealed that a network of electrodes recording from pre-frontal (Fp2), frontal (F4), temporal (T4), parietal (P3, P4), and occipital (O1, O2) scalp regions predicted behavioral performances. Also, the dynamics of ratios from ipsilateral occipital-parietal electrodes were correlated with the changes in performance within the same subject (i.e., between first and second blocks of the same session) independently of task difficulty. Connectivity analysis (as evaluated with Granger causality), indicated that a consistent network of electrodes exchanged information from electrodes located in ipsilateral pre-Frontal (Fp2), contralateral central (C3) and ipsilateral occipital (O2); to ipsilateral pre-frontal (Fp2), ipsilateral temporal (T4), and contralateral parietal (P3) regions. In addition, as subjects improved their performance, less information was exchanged to and from electrode P4, while more information was transferred between electrodes identified in the FP2-C3-T4-P3-O2 network. A summary of these findings is presented in Table 3.

**TABLE 3:** Summary of findings.

	Analysis	N blocks	Period	Significant/relevant electrodes	Function	Electrodes
**Task description**	Power	*N* = 29 (16 subj.)	Disc and Resp	Fp2,Fz-4, T3,C3-z,P3-4, O1-2	Power is different during Disc and Resp	F-C-T-P-O
				More often in theta and alpha		
	Ratios	*N* = 29 (16 subj.)	Disc and Resp	Fp2, F3-4, T3, C3, P3-4, O1	Ratios are different during Disc and Resp	F-C-T-P-O
**Tactile Discrimination**	Ratios vs. % Correct	*N* = 29 (16 subj.)	Disc	Fp2, F4,T4,P3-4, O2	Ratios correlate to performance	F-T-P-O
	Pooled sessions (regular +easy)	*N* = 35 (16+3 subj.)	Disc	Fp2, F4, P3-4	Ratios correlate to performance, independently of task difficulty	F-P
	Ratio dynamics vs. Performance changes	*N* = 28 (14 subj.)	Disc	P4-O2	Ratio 1 dynamics predict changes in performance	P-O
	Pooled sessions (regular +easy)	*N* = 34 (14+3 subj.)	Disc	P4-O2	Ratio 1 dynamics predict changes in performance, independently of task difficulty	P-O
**Information processing**	Granger causality	*N* = 28 (14 subj.)	Disc	Fp2, C3, O2, send to: Fp2, P3, O2, F4, T4	Information is transferred between specific electrodes	Fp2, C3, O2 -> P3, Fp2, T4,F4
	Granger causality	*N* = 28 (14 subj.)	Disc	Increase: Fp2, F4, C3, T4, O2 Decrease: P4, F4, P3	Performance changes with increases/decreases of information transfer	P4(-), P3(+), O2(+)

“Task Description” the electrodes presenting modulations between the discrimination and response periods are presented. “Tactile discrimination” the networks of electrodes that are correlated with performance “between-subjects” and “within-subjects” are presented. “Information processing” the networks associated with Granger causality testing “between-subjects” and “within-subjects” are presented.

The passive tactile width discrimination task requires that subjects insert their index finger in the discriminanda, wait for the bars to touch the finger (tactile discrimination), remove the finger, and then make an appropriate response in a pushbutton (motor response) (Perrotta et al., 2020). Analysis of power during the discrimination and response periods revealed an overall reduction in power occurring more often in theta, alpha, and beta frequency bands (and to a lesser extent in delta and low gamma bands), in a network that included Fp2, Fz-4, T3, C3, P3-4, and O1-2 electrodes. Analysis of ratios of power revealed a network of electrodes that included Fp2, F3-4, T3, C3, P3-4, and O1. These results generally support our first hypothesis (H1) that discrimination and response periods would be associated with changes in power, as expected from previous studies of tactile discrimination (Stilla et al., 2007; Adhikari et al., 2014, Kunicki et al., 2019).

The C3 electrode (which was located in the scalp over the contralateral somatosensory cortex) presented modulation of frequency bands and ratios between the discrimination and the response periods, and was the electrode that presented the highest connectivity in network described here (connecting in 80% of the blocks with Fp2, O2, and P3). Interestingly, its activity did not correlate to task performance. Tactile discrimination has been associated with changes in EEG activity recorded from the electrodes C3/4, namely with the appearance of event related potentials or power changes in alpha and beta frequency bands (Pfurtscheller et al., 2001; Graimann et al., 2002; Spitzer and Blankenburg, 2011; Baumgarten et al., 2015, 2016; Ishigaki et al., 2016; Moungou et al., 2016; Yao et al., 2016, 2017a,b; Genna et al., 2017; Whitmarsh et al., 2017; Eldeeb et al., 2020; Su et al., 2020). For example, a relevant contribution of mu (8–15 Hz) and beta (16–30 Hz) frequency bands has been demonstrated for texture discrimination using a support vector machine classifier in electrodes recording from primary and secondary somatosensory cortex (Eldeeb et al., 2020), and event related synchronization/desynchronization in alpha and beta frequency bands has been used for brain-machine interfaces (Yao et al., 2016, 2017a,b). Our results are partially in line with these studies, to the extent that C3 beta frequency band modulations occurred during the discrimination period. One potential explanation for the discrepancy between our results and previous studies is that the period of tactile stimulation in the tactile width task is relatively long and therefore changes occurring in small timescales may go unnoticed in the present analysis. Another potential explanation is that the relevant tactile processing changes may be occurring in cortical oscillatory dynamics of event related synchronization/desynchronization (Graimann et al., 2002; Yao et al., 2016, 2017a,b) and not in the ratios of frequencies analyzed here.

In addition to beta frequency band, theta band modulation was also present in the C3 electrode. Theta band has been previously associated with tactile function, namely in roughness discrimination (Genna et al., 2017, 2018). Alternatively, such theta modulation could also reflect some form of interaction between the somatosensory and the motor cortex (Shibata et al., 2021) as expected from the change occurring between the tactile discrimination period and the response period (more associated with motor activity). Contrary to previous studies, we have not observed changes in the gamma frequency band for electrode C3. These modulations have been associated with tactile processing in other tactile discrimination tasks (Michail et al., 2016; Jiao et al., 2020; Ross et al., 2022), tactile spatial attention (Bauer et al., 2006), and multisensory communication (Misselhorn et al., 2019). It should be noted, however, that the analysis of ratio 1 during the discrimination and the response periods for the C3 electrode revealed a significant difference, potentially suggesting a role for beta and gamma frequency bands during the discrimination period (also see connectivity discussion below).

Analysis of ratios of frequencies revealed that the transition between the discrimination and the response periods was characterized by an increase in both ratios occurring in electrodes Fp2, F4, T3, C3, and P3-4 electrodes; an increase in ratio 1 for F3 electrode; and an increase in ratio 2 for electrode O1. These results also support our first hypothesis (H1) that the analysis of ratios of frequencies distinguished between the periods of discrimination and response. At this point it is important to note that the results obtained when comparing the ratios of higher and lower frequencies did not simply mimic the analysis performed for the different frequency bands. First, the use of ratios allowed reducing the number of comparisons performed (each ratio already includes at least two frequency bands). Second, the analysis of ratios allows identification of states associated with simultaneous changes occurring in multiple frequency bands (Gervasoni et al., 2004; Pereira et al., 2007; Pais-Vieira et al., 2019). An example of the usefulness of this analysis is that ratios allowed identifying electrodes Fp2 and T4 as being associated with the behavioral performance suggesting that this was due to changes occurring in beta and gamma frequency bands (i.e., the high frequencies that are used to calculate ratio 1). Note that this occurred, even though no significant difference in power between discrimination and response periods was present in these specific frequency bands (Table 1). Therefore, these results suggest that ratios of frequencies can capture small changes occurring in frequency bands that may not necessarily correspond to the classical band divisions (i.e., delta, theta, alpha, beta, gamma) and therefore may not appear in more classical analysis (however, also consider Donoghue et al., 2020).

An association between neural activity during the discrimination period and tactile discrimination performance was found in a bilateral asymmetric network of electrodes spread throughout the scalp (Ratio 1 for electrodes Fp2, F4, T4, P3-4, and O2; and with Ratio 2 for electrodes P3-4). This result supports our hypothesis (H2) that ratios of frequency bands would correlate with tactile performance. These results are partially in line with a previous study where posteromedial parietal cortical activity measured in functional magnetic resonance predicted tactile spatial acuity (Stilla et al., 2007).

### 4.1. Occipito-parietal electrodes

It has been proposed that simultaneous alpha, beta, and gamma frequency band oscillations are required for unified cognitive operations (Misselhorn et al., 2019) such as attention to tactile stimuli (Bauer et al., 2006), and attention to affective touch (von Mohr et al., 2018). The large number of modulations observed across the different frequency bands and electrodes in our study is in line with this previous proposal. For example, decreases in the power of theta and beta frequency bands in the parietal regions during tactile information processing have been associated with attentional and emotional mechanisms (von Mohr et al., 2018), and alpha rhythm, originating from the occipito-parietal areas, is known to be involved in perception, especially in visual attention (Romei et al., 2010). Also, maintaining a previously presented tactile stimulus has been associated with a decrease in alpha and beta frequency bands activity in S1 and an increase in the occipital cortex (Spitzer and Blankenburg, 2011). Our results are partially in line with these previous findings since the activity recorded from electrodes placed in occipito-parietal areas (P3, P4, and O1) presented differences in theta, in beta (P4), as well as in gamma (P3) frequency bands. Also, the activity from electrodes P3, P4, and O2, was correlated to the performance, and the activity in P4 and O2 was correlated to the overall change in performance (i.e., changes between the two blocks). However, we have also found that our modulations frequently presented the opposite pattern of these previous reports. Namely, tactile discrimination was generally characterized by increased power in alpha (P3) and beta (P4) frequency bands, when compared to the response period. It is not clear at this point if this is related to the different strategies that subjects use during the task, or otherwise if this may be the result of the multiple cognitive and sensorimotor activities required to complete the task (sampling, waiting for the lights to change, choosing between different pushbuttons, etc.). Altogether, these results suggest that fronto-parietal electrodes are associated with task execution while neural activity from pre-frontal, temporal, and occipital electrodes may play a significant role solely when high levels of attention and/or learning were required.

The dynamics of ratio 1 in electrodes P4 and O2 predicted the amount of change in behavioral performance for the different blocks of a given subject. This finding partially supports our hypothesis that changes in performance in the same subject would be associated with changes in neural activity recorded from frontal, temporal, parietal, and occipital electrodes (H2). It has been previously argued that task difficulty may be related to attention, engagement, arousal as well as other constructs or properties (Pope et al., 1995; Brouwer et al., 2012; Heard et al., 2018; Faller et al., 2019; Jao et al., 2020), and that lateralized activity in alpha frequency band recorded from sensorimotor regions may be a relevant neurophysiological feature of difficulty levels (Jao et al., 2020). Our additional experiments, with a small sample of subjects performing an easier version of the task, suggested that the neural correlates of these dynamics were independent of task difficulty. Connectivity analysis revealed that the occipital electrode O2 received information from all electrodes, but more consistently from C3 (contralateral), as well as ipsilateral pre-frontal, frontal, and temporal electrodes (Fp2, F4, T4, P4). This relevant role for the occipital electrode, especially in a task where the subject cannot observe the stimulus delivered, seems to be in line with previous reports of humans performing tactile discriminations (Zangaladze et al., 1999; Stilla et al., 2007; Adhikari et al., 2014), and also with a previous report on the relevant role of the occipital cortex in rodents performing a tactile width discrimination task in the dark (Kunicki et al., 2019).

The P4 electrode participated in both networks described here (“between-subjects” and “within-subjects”) and, except for an increase to and from electrode O2, an overall reduction in connectivity to this electrode occurred between the two blocks (also see Supplementary Table 2). Activity in the parietal cortex is known to be related to attention to tactile stimuli (Colby and Goldberg, 1999; Pasalar et al., 2010; Ku et al., 2015a,b; Deschrijver et al., 2016). This is in line with the finding of high connectivity between contralateral P3, C3, F4, and T4. Meanwhile, this reduction in connectivity was directed to and from to T4, P4, and P3, and occurred in a large number of blocks. We hypothesize that this finding may reflect an overall reduction in attention levels occurring as subjects become more proficient in the task (Colby and Goldberg, 1999, Pasalar et al., 2010; Ku et al., 2015a,b; Deschrijver et al., 2016). An alternative explanation, however, could be related to the time allowed for the subject to remain with the finger in the tactile stimulation chamber (recall that, after the tactile stimulus delivery, a red light turns on in the front panel of the box indicating that subjects must remove their finger and make a response). For example, it is possible that unexperienced subjects may be facing some form of conflict as they are trying to maintain the finger in the discriminanda for the largest amount of time possible but are required to make a response. If this alternative explanation is correct, then neural activity in this electrode could potentially be related to some form of movement inhibition (Hannah and Jana, 2019; Osada et al., 2019). Independently of the correct explanation, our findings suggest an asymmetrical role for parietal electrodes in regards to information transfer.

### 4.2. Networks for information processing

The present results are partially in line with previous studies analyzing networks involved in tactile processing. Stilla and colleagues have reported that tactile processing was associated with a network involving frontal-parietal-occipital areas (Stilla et al., 2007). These authors demonstrated that performances could be predicted from nodes of this network and, in addition, that the right posterior intraparietal sulcus was a relevant node of convergence of information from other regions. In the present study, electrode P4 was associated with changes during the discrimination and response periods and was correlated with task performance in both networks described (“between-subjects” and “within-subjects”), therefore supporting these previous findings. However, our analysis of connectivity suggests that, for tactile width discrimination, transfer of information to and from this electrode (with the exception of O2, see Figure 4) may either have the opposite effect and impair performance, or otherwise reflect some form of increased attention levels or conflict, as discussed above.

In a more recent study, Adhikari et al. (2014) have also described a network involving the contralateral primary somatosensory cortex, the ipsilateral occipital cortex, the right posterior parietal cortex, and the contralateral pre-frontal cortex, that communicated through beta and gamma frequency bands. As discussed above, the network of regions described in our study is also partially in line with the findings from this previous study, even though we have found no relevant role for the contralateral pre-frontal (Fp1) and frontal electrodes (F3). One potential explanation is that this network operated in a much smaller time scale (less than 200 ms) than the networks reported by us. Altogether, the results from the present study are generally in line with previous studies in respect to the main nodes and frequency bands relevant for tactile information processing. Meanwhile, the details of specific frequency bands and information transfer between electrodes will require additional studies.

### 4.3. Comparison to rodents

In a previous study in rodents, we have found that width discrimination was associated with information transfer in a fronto-parieto-occipital network (Kunicki et al., 2019). Here, we demonstrated–in humans–that a fronto-parieto-occipital network was also involved in width discrimination and, in addition, that different functions could be associated with two sub-networks. Namely, that the fronto-parietal network was associated with tactile width discrimination performance between-subjects (interspecific), while a parieto-occipital network was associated with the changes in performance within-subjects (intraspecific). The present findings, therefore, suggest a path for new experiments in rodents, either by directly activating or silencing critical regions of these networks during tactile width discrimination, or by altering the characteristics of the behavioral task (for example, introducing multiple blocks of trials). In addition, the present study also suggests that networks associated with “between-subjects” and “within-subjects” processing, should have different subcortical substrates (e.g., involving limbic structures and/or thalamic nuclei) (Banerjee et al., 2023). Therefore, the findings of the present study support the notion that a systematic description of tactile width discrimination in humans can significantly improve our current knowledge on tactile processing in this species and benefit from the large body of knowledge existing in rodents (Krupa et al., 2001, 2004; Wiest et al., 2010; Pais-Vieira et al., 2013a,b, 2015; Thomson et al., 2014; Kunicki et al., 2019), while also contributing with new research directions.

### 4.4. Caveats, potential bias, and technical discussion

A small number of caveats and potential bias should be considered. The passive version of the width discrimination task uses two movable bars that touch the index finger of the user. This means that not only the aperture width changes in each tactile stimulus, but also the pressure exerted by the bars in the index finger. It will be important to determine, in subsequent studies, to which extent neural correlates of pressure discrimination differ, or not, from neural correlates of width discrimination.

The number of electrodes used to record in this study does not allow for source analysis and therefore, the present findings are discussed regarding the position of each electrode rather than the cortical region beneath it. Analysis of ratio 1 in this study included low (30.5–45.0 Hz), but not the high gamma band frequencies which are relevant for tactile spatial attention (Bauer et al., 2006). We have opted for this because: (i) visual inspection of the data showed little activity in the higher gamma band, and (ii) we have previously observed that these specific ratios seem to capture well the dynamics of tactile information processing in rodents, especially when analyzing longer periods (Pais-Vieira et al., 2019). Even so, the present results and conclusions can be, to some extent, biased by this approach.

Another relevant caveat is that the number of samples compared in the analysis of between- and within-subjects is different. This occurred due to the experimental design, as well as due to technical problems and therefore, some of the present results should be taken cautiously.

The analysis of results obtained from the ratio 1 (i.e., higher frequencies) of power should be also considered with some caution. The original manuscript that described this technique (Gervasoni et al., 2004) reported that these intervals were chosen because it was found that they best separated the different types of behavior observed in rodents. However, both ratios (ratio 1: higher frequencies, and ratio 2: lower frequencies) are not mutually exclusive and include the lower frequencies. In future studies, it will be important to describe and model in detail how changes in each of the different frequencies can affect the resulting ratio.

Lastly, for one of our subjects (S2), performing the task proved to be a stressful event. Such an effect of the task was not predictable from our previous (Perrotta et al., 2020) or current experience (now including more than 30 subjects tested in different versions of the task), since subjects are always allowed to interact with the task before the session (i.e., they are allowed performing a small number of trials until they indicate being comfortable with the procedure). Even though, in future studies, we propose implementing a short psychological evaluation of subjects before testing them in this apparatus.

### 4.5. Conclusion

Tactile width discrimination was associated with changes in neural activity and connectivity in networks involving electrodes from fronto-temporo-parieto-occipital networks, mostly in theta, alpha, and beta frequency bands. Asymmetrical networks of electrodes were associated with tactile width discrimination performance within- and between-subjects. These results represent the first detailed description of EEG activity during a tactile width discrimination task and expand previous findings on the widespread asymmetrical involvement of cortical networks during tactile width discrimination.

## Data availability statement

The datasets presented in this study can be found in online repositories. The names of the repository/repositories and accession number (s) can be found below: https://osf.io/sbnqe/?view_only=6634716552c74f5aa6d1cf64d693fd10.

## Ethics statement

The studies involving human participants were reviewed and approved by the Ethics Committee of the University of Minho (SECVS 148/2016) and the Comité para as Ciências da Saúde of the Catholic University of Portugal (39/2017). The patients/participants provided their written informed consent to participate in this study.

## Author contributions

CP-V, MKA, AP, and MP-V collected the data. CP-V, MO, CK, ASP, MA, and MP-V analyzed the data. All authors participated in the manuscript writing and agreed to the final version of the manuscript.
