# Supplementary material for: Neurophysiological correlates of tactile width discrimination in humans
Source: Front Hum Neurosci. 2023 May 12;17:1155102. doi: 10.3389/fnhum.2023.1155102 (PMC10213448; doi:10.3389/fnhum.2023.1155102)
Supplement: Supplementary file 1 [file Data_Sheet_1.PDF]

Supplementary Material

1 Supplementary Tables

	Fp1	Fp2	F3	Fz	F4	T3	C3	Cz	C4	T4	P3	Pz	P4	O1	O2	Tp10
Fp1	0	0	0	0	0	0	0	0	0	0	0	0	0	0	0	0
**Fp2**	0	0	0	0	57.1	0	71.4	0	0	85.7	64.3	0	57.1	0	78.6	0
F3	0	0	0	0	0	0	0	0	0	0	0	0	0	0	0	0
Fz	0	0	0	0	0	0	0	0	0	0	0	0	0	0	0	0
**F4**	0	64.3	0	0	0	0	57.1	0	0	64.3	57.1	0	50	0	64.3	0
T3	0	0	0	0	0	0	0	0	0	0	0	0	0	0	0	0
**C3**	0	78.6	0	0	64.3	0	0	0	0	64.3	78.6	0	50	0	78.6	0
Cz	0	0	0	0	0	0	0	0	0	0	0	0	0	0	0	0
C4	0	0	0	0	0	0	0	0	0	0	0	0	0	0	0	0
**T4**	0	71.4	0	0	64.3	0	71.4	0	0	0	64.3	0	50	0	71.4	0
**P3**	0	57.1	0	0	71.4	0	71.4	0	0	64.3	0	0	57.1	0	50	0
Pz	0	0	0	0	0	0	0	0	0	0	0	0	0	0	0	0
**P4**	0	50	0	0	64.3	0	57.1	0	0	64.3	50,0	0	0	0	57.1	0
O1	0	0	0	0	0	0	0	0	0	0	0	0	0	0	0	0
**O2**	0	50	0	0	78.6	0	64.3	0	0	71.4	64.3	0	71.4	0	0	0
Tp10	0	0	0	0	0	0	0	0	0	0	0	0	0	0	0	0

Supplementary Table 1. Proportion of significant Ganger causality tests between specific pairs of electrodes. Tests were only performed between electrodes Fp2, F4, C3, T4, P3, P4, and O2 for the 14 subjects that performed both blocks. Electrodes in rows send information to electrodes in columns. For example, electrode Fp2 sent information to electrode T4 in 12 blocks (12/14=85.71%) and the T4

electrode sent information to the electrode Fp2 in 10 blocks (10/14=71.43%). The zeros indicate that the pair was not tested.

	Fp1	**Fp2**	F3	Fz	**F4**	T3	**C3**	Cz	C4	**T4**	**P3**	Pz	**P4**	O1	**O2**	Tp10
Fp1	0	0	0	0	0	0	0	0	0	0	0	0	0	0	0	0
**Fp2**	0	0	0	0	1	0	1	0	0	2	2	0	1	0	3	0
F3	0	0	0	0	0	0	0	0	0	0	0	0	0	0	0	0
Fz	0	0	0	0	0	0	0	0	0	0	0	0	0	0	0	0
**F4**	0	-1	0	0	0	0	1	0	0	-1	4	0	-3	0	3	0
T3	0	0	0	0	0	0	0	0	0	0	0	0	0	0	0	0
**C3**	0	1	0	0	3	0	0	0	0	-1	3	0	-1	0	1	0
Cz	0	0	0	0	0	0	0	0	0	0	0	0	0	0	0	0
C4	0	0	0	0	0	0	0	0	0	0	0	0	0	0	0	0
**T4**	0	2	0	0	1	0	2	0	0	0	3	0	-3	0	-2	0
**P3**	0	1	0	0	3	0	2	0	0	1	0	0	-3	0	-2	0
Pz	0	0	0	0	0	0	0	0	0	0	0	0	0	0	0	0
**P4**	0	1	0	0	-4	0	0	0	0	-3	-4	0	0	0	2	0
O1	0	0	0	0	0	0	0	0	0	0	0	0	0	0	0	0
**O2**	0	3	0	0	1	0	3	0	0	-2	-1	0	3	0	0	0
Tp10	0	0	0	0	0	0	0	0	0	0	0	0	0	0	0	0

Supplementary Table 2. Number of sessions where increases or decreases in Granger causality tests were found between the first and second blocks. Tests were only performed between electrodes Fp2, F4, C3, T4, P3, P4, and O2 for the 14 subjects that performed both blocks. Electrode P3 stopped sending information to electrode P4 in 3 blocks (indicated as -3 blocks without significant Granger causality tests), meanwhile P3 started sending information to F4 (indicated as +3 blocks with significant Granger causality tests). The largest decreases occurred between electrodes P4 and F4, and electrodes P4 and P3 (both with a decrease of -4). The largest increase occurred between electrodes F4 and P3 (+4). Zeros indicate that pairs not tested.
